# Temporal and Spatial Detection of the Onset of Local Necking and Assessment of its Growth Behavior

**DOI:** 10.3390/ma13112427

**Published:** 2020-05-26

**Authors:** Christian Jaremenko, Emanuela Affronti, Marion Merklein, Andreas Maier

**Affiliations:** 1 Pattern Recognition Lab, Friedrich-Alexander-Universität Erlangen-Nürnberg Martensstr. 3, 91058 Erlangen, Germany; andreas.maier@fau.de; 2 Institute of Manufacturing Technology, Friedrich-Alexander-Universität Erlangen-Nürnberg Egerlandstr. 13, 91058 Erlangen, Germany; emanuela.affronti@fau.de (E.A.); marion.merklein@fau.de (M.M.)

**Keywords:** pattern recognition, machine learning, deep learning, classification, segmentation, artificial intelligence, forming limit curve, sheet metal forming

## Abstract

This study proposes a method for the temporal and spatial determination of the onset of local necking determined by means of a Nakajima test set-up for a DC04 deep drawing and a DP800 dual-phase steel, as well as an AA6014 aluminum alloy. Furthermore, the focus lies on the observation of the progress of the necking area and its transformation throughout the remainder of the forming process. The strain behavior is learned by a machine learning approach on the basis of the images when the process is close to material failure. These learned failure characteristics are transferred to new forming sequences, so that critical areas indicating material failure can be identified at an early stage, and consequently enable the determination of the beginning of necking and the analysis of the necking area. This improves understanding of the necking behavior and facilitates the determination of the evaluation area for strain paths. The growth behavior and traceability of the necking area is objectified by the proposed weakly supervised machine learning approach, thereby rendering a heuristic-based determination unnecessary. Furthermore, a simultaneous evaluation on image and pixel scale is provided that enables a distinct selection of the failure quantile of the probabilistic forming limit curve.

## 1. Introduction

The forming range of sheet metal to produce defect-free components is evaluated by means of the forming limit curve (FLC), whose limits are defined by the major and minor strain pairs and define the onset of local necking immediately. In Europe, the method for generating the FLC is summarized in DIN EN ISO 12004-2 [1], whereby the FLCs are generated with the help of Nakajima [2] and Marciniak [3] test set-ups. In this process, the sheet is mounted in a clamping unit and then formed by a flat-shaped or hemispherical punch until fracture. The evaluation method proposed in the ISO standard is based on the Bragard study of 1972 [4], which is referred to as the “cross-sectional method”, and evaluates the strain distribution of the material before fracture. For this purpose, cross sections perpendicular to the crack initiation are defined and the limit strains are approximated by means of the last step of the strain distribution using a second order polynomial. Nowadays, the strain progression is also taken into account, so that the forming behavior and the FLC are determined using digital image correlation (DIC) methods [5]. In this regard, the forming experiments are recorded with a stereo camera system and the strains on the material surface are determined, in order to facilitate the evaluation of the strain development. Despite these technological advances, the standard is still based on the “cross-sectional method”, where the strain development is not taken into account for the determination of the FLC. In addition to the technological aspects, the intrinsic properties of the materials also play a major role in determining the deformation limits. For example, modern lightweight materials such as high-strength steels and aluminum alloys tend to develop several local strain maxima or fracture spontaneously without a necking phase. Consequently, the second order functions proposed in the standard are not suitable for approximation of the limit strains. To overcome the limitations of the “cross section method”, different methods have been proposed which take into account the forming progression during Nakajima tests. These time-dependent methods include the “line-fit” method [6] and the correlation coefficient method [7]. Both approaches are based on the observation of thickness reduction within a predefined zone of instability and the detection of the onset of necking in reaction to a sudden decrease in thickness direction of the sample. In addition to these established methods, further approaches have been proposed that are based on heuristics, which compare the surface height difference between a point within the necking location to a point of the homogeneous deformation area [8], assess two user determined evaluation areas of different radii that include the necking area [9], or identify a flattening process and valley development based on the z-displacement and its spatial derivative [10]. All methods mutually share the drawback of a predefined instability zone or well-considered area of evaluation.

In order to derive new insights and concepts for forming development, a method based on machine learning was proposed in 2015 [11]. In principle, pattern recognition addresses the automatic processing and assessment of data [12], whereby physical signals, e.g., sensor data or images of a video sequence, are first transformed into characteristic features that have been defined by experts of the respective field. In order to facilitate an automated separation of the data into subspaces/classes, the data is initially divided into different classes by experts for supervised machine learning procedures, whereby each data point is assigned a class label. Subsequently, a classification algorithm is used to learn decision boundaries based on the feature representations/label pairs with a representative subset of the data. Afterwards, the generalization of the separation hypothesis or the learned class boundaries is evaluated with the remaining disjoint data. As demonstrated in metallographic investigations, both conventional deep drawing steels [13] and dual-phase steels [14] exhibit superficial patterns that correlate with local necking and are also prominent in the strain distributions. These findings were first exploited in a supervised machine learning process using the strain distributions together with appropriate features [15]. Affronti et al. [16] adopted the above mentioned approach and extended it to a four class problem (homogeneous forming, diffuse necking, local necking, and crack) and contrasted the classification results with the expert annotations for the different failure categories. This study demonstrated that a consistent determination of local necking is possible with the help of expert knowledge and machine learning. Additionally, it was concluded that the accuracy of the results is affected by subjective expert annotations and correlating external factors such as the sampling frequency. In order to eliminate the dependence on expert annotations, an unsupervised classification approach was proposed in [17]. This approach evaluates the region of local necking using a histogram of oriented gradients (HoG) [18] to classify the onset of local necking using one-class support vector machines [19]. It was demonstrated that the results are within the range of the “line-fit” method, whereby a specific definition of the exact necking area is omitted as a result of larger evaluation regions. The unsupervised approach not only introduced an independence from expert annotations, but also a probabilistic FLC that enables the determination of different forming limits with respect to the failure likelihood. However, one disadvantage of this method is the relative dependence on the evaluation position, as the evaluation regions are defined depending on the strain maximum. Additionally, the criteria for the determination of local necking based on the HoG characteristics are limited by the prior knowledge of the experts, so that a generalization to other materials is again not guaranteed. Furthermore, the criteria for the determination of local necking based on the HoG characteristics is limited by a priori knowledge of the experts, so that a generalization to other materials is not guaranteed. In recent years, convolutional neural networks (CNN) have become widely useful in the field of pattern recognition. As a consequence of technological advances, it is now feasible to process large amounts of data in finite time. The main advantage of these approaches is the automated, data-driven learning of representative features adapted to the problem, eliminating the need for a priori knowledge for handcrafted feature representation. In several competitions, data-driven CNN approaches have outperformed conventional feature based pattern recognition methods [20], such that they are now successfully used in a wide range of applications. In the field of medical technology, these include non-rigid registration [21], reconstruction [22], landmark recognition [23], and in mechanical engineering, event detection [24], or defect detection for photovoltaic module cells [25].

**Figure 1:**
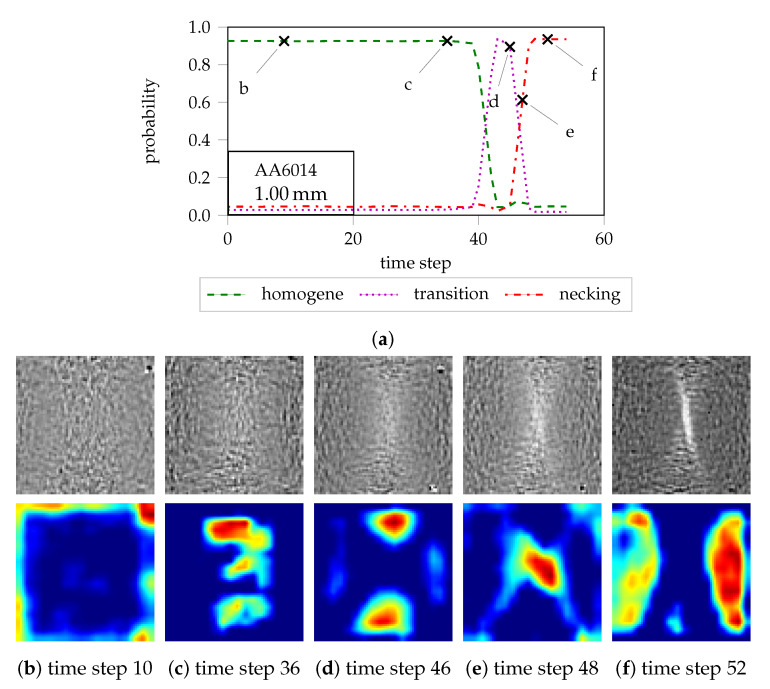
The temporal progression of cluster memberships for one forming sequence with strain difference images and corresponding classification heat maps at different time-points: (**a**) Cluster membership progression for AA6014-S050-1 with three different forming phases according to the work in [26]. (**b**–**f**) illustrate the difference images and class activations of the homogeneous forming phase at time-point 10 and 36, the transition phase at time-point 46, the necking phase at time-point 48, and the end of the forming process or necking phase at time-point 52.

For the determination of local necking, Jaremenko et al. [26] introduced a semisupervised approach based on deep learning. This approach employs the incremental changes between successive strain distributions as suggested by Vacher et al. [27] and comprises two steps: In the first step, optimal features are learned in a supervised classification manner using images of the beginning of the homogeneous forming phase and images of the end of the inhomogeneous forming phase near the failure of the material. Based on a Siamese network topology [28], representative features for these two classes are simultaneously learned and separated optimally within the feature space. The second step incorporates a clustering procedure using Student t Mixture Models (SMM) [29]. As a result of the mixture models, an assignment of the individual frames to the respective clusters and thus a probabilistic evaluation is possible. Even though the features were learned with only two classes, a three-class classification of the forming process into a homogeneous, transition and inhomogeneous forming phase is provided by the cluster method (cf. Figure 1a). An advantage of this methodology is the complete independence from time and predefined evaluation area. This enables the generation of failure probabilities for strain paths, even though the specimens have not been formed to fracture. Additionally the definition of a necking region is not required. Another important factor is the possible application of the well-established Grad–Cam method [30]. This approach highlights the relevant regions for the decision process, i.e., from the network point of view, by providing a heat map for each strain distribution of the forming sequence, thus facilitating visual interpretation of the results and the determination of the onset of necking. As an example, Figure 1 visualizes the cluster membership progression for AA6014-S050-1 with three different forming phases (cf. Figure 1a as suggested in [26]) together with the strain difference images and their corresponding class activation maps. At time-point 10 (cf. Figure 1b), the difference image is characterized by a homogeneous strain distribution and class activation map. Another example for the homogeneous forming phase is provided in Figure 1c for time-point 36, where a slight concentration of strain is now visible within the center of the image. However, the class activations are distributed among the whole image without a clear focus on the central part. As the process enters the transition phase at time-point 46 (cf. Figure 1d), instead of focusing on the central occurring structure, the class activations maps highlight the triangular regions above and below this area. With further forming, the focus on the central structure or concentration of strain occurs at time-point 48 (cf. Figure 1e) with the emerging localized necking and beginning of the necking phase. Subsequently, towards the end of the forming process, the class activations emphasize the area besides the central strain concentration (cf. Figure 1f).

As depicted in Figure 1e, the beginning of local necking is represented by the focus on the central structure. However, only a coarse necking area is identified, so that an exact determination at the level of a single pixel that contributes to the necking effect is not possible. Similar to the other studies and methods without heuristics, this coarse approximation prevents the identification of the actual necking area and the characterization of the development of the necking region during forming processes.

In order to improve the general understanding of the local necking area and its emergence, expansion, and evolution, this study extends the approach of Jaremenko et al. [26]. Likewise, by means of a semisupervised approach, a high-resolution determination of the necking area without heuristics is proposed. Consequently, a simultaneous assessment of the necking effect, at both, image and pixel scale, is provided which facilitates the analysis and definition of the necking area. These findings therefore improve the interpretability of the probabilistic FLC, facilitating the selection of failure quantiles with improved precision.

## 2. Test Set-Up of the Forming Experiments and Materials

The FLC is usually determined experimentally with a Nakajima test set-up [2], coupled with an optical measurement system comprising a stereo camera setup, a clamping unit with an inner die diameter of 110 mm and a hemispherical punch with a diameter of 100 mm. In order to determine the strain values and distributions, the optical measuring system (ARAMIS GOM GmbH, Braunschweig, Germany) is used to record a forming process that is post processed by means of a DIC algorithm [5]. A requirement for the calculations of correlations and displacements is the preparation of the specimens with a white primer and a speckle pattern of black graphite. A lubrication system according to DIN EN ISO-12004-2 [1] is used to minimize the friction between the punch and the specimen such that a rupture is initiated at the top of the specimen. The sample geometries to be investigated are varied throughout the test procedure, starting from conical blanks with parallel connections up to an uncut sample geometry, so that different loading conditions and strain paths are initiated. The name of the sample geometry is determined by the remaining conjunction width, e.g., 50 mm corresponds to S050. In order to evaluate different boundary conditions and to test the ability of the method to generalize to new material, the test parameters such as punch speed (1 to 2 mm/s) and sampling rate (15, 20, and 40 Hz) are varied. Overall, this study investigates three materials: (1) DX54D, a deep drawing steel, with ductile necking behavior and observable localization on the surface; (2) DP800, a dual-phase steel, high-strength material with a matrix of ferrite and martensite precipitations and hence multiple observable local maxima during Nakajima tests; and (3) AA6014, a lightweight aluminum alloy of the 6xxx series with multiple maxima during Nakajima tests under plain strain conditions. An overview of the principal material properties in terms of the hardening exponent (n), yield strength (YS), tensile strength (TS), uniform elongation (UE), and the Lankford coefficients (r_0_, r_90_) are provided in Table 1 for the three different materials.

## 3. Machine Learning Methodology

The proposed method essentially follows the typical processing pipeline used in pattern recognition solutions despite the use of a CNN approach. This pipeline comprises four steps, (1) data acquisition, (2) preprocessing, (3) feature extraction, and (4) classification. The main difference compared to the conventional development of pattern recognition solutions is the data-driven feature extraction and its combination with the classification step by means of deep learning techniques. Consequently, the feature extraction and classification steps are combined and designed as an optimization problem, instead of relying on predefined, handcrafted features and a classification algorithm to be selected. By solving the optimization problem an estimation of a problem-specific optimal solution is approximated, which is realized, e.g., by minimizing a suitable problem-specific loss function. For this purpose, the data set is typically subdivided into several disjoint subsets. While training the network, the loss function is minimized using a training data set to adjust the parameters of the individual layers of the CNN gradually, using a gradient descent optimizer. In a sequential procedure, all training data is repeatedly passed to the network, whereby a complete pass of the training data is referred to as an epoch. At the end of each epoch, the separation hypothesis is evaluated with a validation data set. Overall, this procedure is repeated until convergence of the optimization function. Afterwards, the separation hypothesis is assessed with a disjoint test dataset to verify whether the learned hypothesis can generalize to unseen data. Similar to conventional pattern recognition approaches, CNN provides supervised and unsupervised procedures, requiring either expert labels or resolving the optimization problem without those expert annotations.

In general, our proposed method closely follows the concept of the weakly supervised approach of Jaremenko et al. [26] that comprises two steps: (1) the supervised feature learning part, which ensures that the instances of the homogeneous and inhomogeneous forming phase are optimally separated from each other within feature space, and (2) the unsupervised cluster procedure by means of SMM to assign the individual images of a complete forming process to the three sub-phases, consisting of homogeneous, transition, and inhomogeneous forming phases. The focus of our approach is the differentiation of the individual forming phases on image scale, with respect to the image characteristics of the entire image without focusing or emphasizing explicitly occurring structures.

This is exactly where our suggested method comes into play, so that fine structures that are characteristic for material failure are segmented early within forming sequences such that the development of the critical area can be accurately traced on a pixel scale. For this purpose, small changes are made to the network architecture and additional information is provided during the feature learning part by means of segmentation masks, so that essentially only the optimization function requires adjustment. The proposed method therefore has two essential properties. On the one hand, an evaluation of the local necking at image scale is provided by the two-step methodology according to Jaremenko et al. [26], and, on the other hand, the architectural changes simultaneously enable an evaluation of the necking at pixel scale. This improves the interpretation of local necking so that an appropriate failure quantile of the probabilistic FLC can be selected based on the characteristics of the local necking at pixel scale, additionally, to the distance quantity at image scale. In the following, the approach of the determination of the FLC at image scale according to Jaremenko et al. [26] is first introduced, followed by the modification for the determination of the necking at pixel scale. Note that an evaluation can be performed exclusively at pixel scale, so that the feature learning and unsupervised clustering could be omitted. However, as a consequence, an FLC can no longer be generated and therefore only an analysis of the necking area would be possible.

### 3.1. Preprocessing

**Figure 2:**
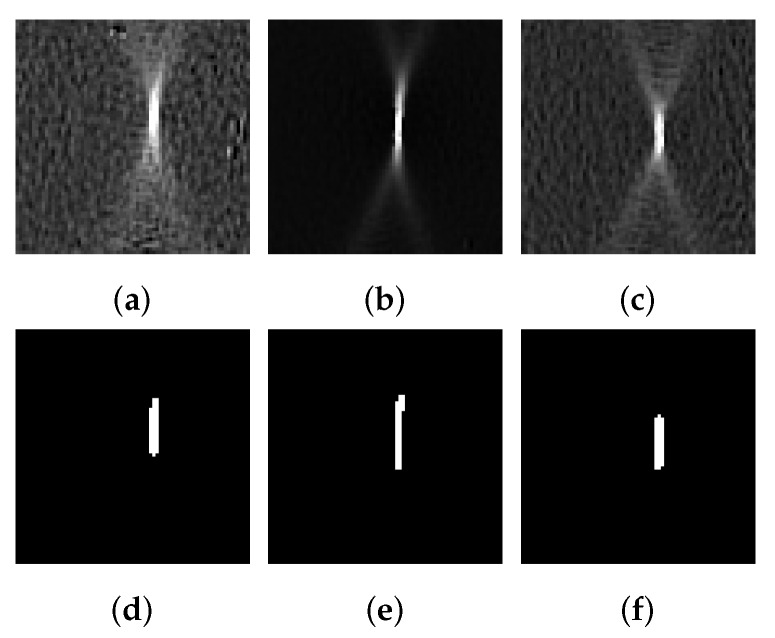
Difference strain images of DP800-S050-(1-3) (**a**–**c**) together with their corresponding segmentation masks (**d**–**f**). The black and white segmentation masks represent the corresponding parts of the difference strain images that characterize the local necking effect. White pixels indicate a contribution to the necking effect, whereas black pixels are considered as background.

The study uses the data set from the work in [17], which was generated using the optical measurement system ARAMIS (v6.3.0-7). Accordingly, the individual forming experiments comprise three-channel video sequences consisting of major and minor strain as well as thinning. In accordance with [17], the differences between two successive images are again employed as input. On the one hand, this removes the correlation with the punch displacement and on the other hand emphasizes the incremental strain differences so that the effect of local necking can, potentially, be better observed. In order to facilitate processing and to avoid unreliable information, individual images were removed from the forming sequences. These are commonly occurring towards the end of the forming process and are characterized by an increasing number of defect pixels, which are increasingly prevalent due to specimen cancellation and thus DIC failure. Occasionally, missing pixel values are linearly interpolated using the temporal information, i.e., taking into account its individual strain progression. Static defect pixels that contain no valid information throughout the forming process are substituted by the average value of a 3 × 3 neighborhood. In order to train the network independently of the different sample geometries and thus different image resolutions, a rectangular area with a side length of 72 px is centrally cropped from the original sequences (cf. Figure 2a–c), so that the same evaluation area is available for all materials and geometries. In contrast to the previous preprocessing methods (cf. [26]), a robust scaling of the data is employed in this study. Outliers are therefore not removed from the data or truncated with a saturation value (0.0 or 1.0), so that the intensity values are only approximately in the range of [0,1]. This procedure is essential to preserve the character of the local necking and to facilitate segmentation. For difference images, the local necking can be identified as a local maximum with falling flanks or metaphorically described as a mountain summit. Thus, a robust scaling with the percentiles 0.25 and 99.75 is used for each frame, so that the majority of the distribution is scaled approximately in the range of 0 to 1 without limiting the minimum and maximum values. This step is extremely important because the intensity distribution is highly skewed and the top end of the gray value range of interest partially overlaps with outliers that are caused, for example, by non-regular measurement errors. In the absence of outliers, previous preprocessing methods [26] would thus replace the maximum value in the region of interest, which might result in a loss of the conical character and consequently might lead to a plateau.

### 3.2. Network Architecture

The proposed method adopts the Siamese architecture of the weakly supervised methodology [26] to enable the evaluation on image scale. This network consists of two identical subnetworks, each using the first three convolution blocks of a VGG16 network [31] pre-trained on ImageNet database [32]. In order to adapt to the new data, an additional dropout layer (0.5 dropout rate) was inserted between the two fully connected layers (512 and 256 neurons), which was followed by an L2 normalization layer. Consequently, this network architecture transforms an input image with a resolution of 72×72 px within the so-called encoding path to a representative feature vector of size 256 at the output of the L2 normalization layer, the so-called bottleneck layer. Until this point, the architecture is strictly adopted from the work in [26]. In this study, an additional decoding path is added to realize the evaluation on pixel scale. This decoding path mirrors the structure of the encoding path with an additional softmax layer as final layer to enable pixel-wise probabilistic class affiliation. Therefore, starting with the bottleneck, an output image according to the size of the input image on the basis of the feature vector is generated. Additionally, in order to emphasize finer structures, the features of the convolutional layers preceding the max-pooling layers of the encoding path are added to the corresponding layer in the decoding path. These so-called skip connections enhance the segmentation of fine structures by using the high-resolution features of the encoding path [33], so that basically a conceptually similar network architecture to the well-known U-Net is created [34].

### 3.3. Feature Learning

**Figure 3:**
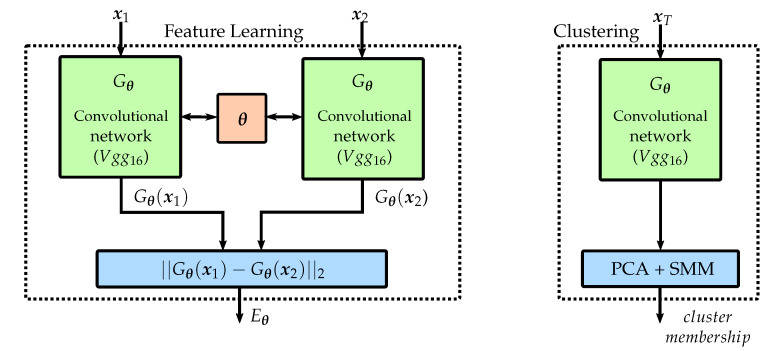
Supervised feature learning and unsupervised clustering. Source: [26] (CC BY 4.0).

The two CNNs form a Siamese CNN architecture [28] and encode the individual frames of a forming sequence to low-dimensional feature vectors, which are then used to evaluate the similarity in a pairwise manner. In order to realize this comparability, two input images ($x_{1},x_{2}$) are evaluated using the identical network structure ($G_{\theta }$) with shared parameters (θ), to facilitate a comparison between the L2 normalized feature vectors ($G_{\theta }\left(x_{1}\right)$,$G_{\theta }\left(x_{2}\right)$) by means of a distance metrics (cf. Figure 3).

The loss function employed in this study evaluates the performance between pairs of inputs $x_{1}$, $x_{2}$, while learning the parameterized distance function $E_{\theta }$ based on the Euclidean distance between the low-dimensional outputs of $G_{\theta }$.

(1) $$E_{\theta }\left(x_{1},x_{2}\right)={\left|\left|G_{\theta }\left(x_{1}\right)-G_{\theta }\left(x_{2}\right)\right|\right|}_{2}$$

The overall loss function is composed of two losses that penalize the model differently, whether the pairs of examples are from the similar ($L_{S}$) or different ($L_{D}$) class, and is described as

(2) $$L\left(\theta \left(y,x_{1},x_{2}\right)\right)=\left(1-y\right)L_{S}\left(E_{\theta }\left(x_{1},x_{2}\right)\right)+yL_{D}\left(E_{\theta }\left(x_{1},x_{2}\right)\right)$$

where $\left(y,x_{1},x_{2}\right)$ denotes a labeled input sample pair. The label *y* refers to the extreme cases of the input sequence, where y=0 if the samples are from the same class and y=1 otherwise. The final loss function, the contrastive loss [35], is denoted as

(3) $$C_{CL}\left(\theta_{e},y,x_{1},x_{2}\right)=L\left(\theta ,y,x_{1},x_{2}\right)=\left(1-y\right)\frac{1}{2}{\left(E_{\theta }\left(x_{1},x_{2}\right)\right)}^{2}+\left(y\right)\frac{1}{2}{\max(0,m-E_{\theta }\left(x_{1},x_{2}\right))}^{2}$$

where the subscript $\theta_{e}$ denotes that only the parameters of the encoding path are modified. The margin parameter *m* specifies a radius around $G_{\theta }\left(x\right)$, the threshold distance for dissimilar pairs. This parameter cannot be optimized due to the incompleteness of the data, as only extreme cases are used for training and thus is set to 1.0 naively. Overall, the individual losses are designed such that minimizing the contrastive loss encourages the model to output feature vectors that are more similar for low values of $E_{\theta }$, whereas if the classes are different, high values of $E_{\theta }$ will lead to less similar feature vectors. Consequently, the data is separated in the latent space by reducing the intra-class variations and maximizing the inter-class distances, without directly enforcing this condition.

### 3.4. Unsupervised Clustering

So far, the network has learned to generate low-dimensional feature vectors that provide an optimal separation of the extreme cases of the forming phases. By encoding entire forming sequences, features are derived for each frame, which can be attributed to the individual failure states (homogeneous, transition, and inhomogeneous) using clustering methods. In order to facilitate the unsupervised clustering process, a dimensional reduction is employed by means of principal component analysis (PCA). The number of components is selected such that more than 95% of the variance of the data is covered, which is consistently attained with two components. In order to avoid sparsely populated regions and to increase the number of instances available for clustering the data, the sample density is increased by artificially augmenting the data. This means that each image of the hold-out test set is randomly cropped, rotated (15 degrees), translated (5 px), and mirrored (horizontal and vertical). The impact of such transformations on the feature space is well described and visualized in [35]. In the course of the evaluation, the loading conditions and thus the geometries of the hold-out material are assessed separately. Therefore, for each geometry, three sequences ($x_{T}$) are encoded by the network into their low-dimensional representations. After applying the PCA-dimensionality reduction, the data is clustered with SMMs [29] to obtain the individual distributions. This enables the determination of a cluster membership for each individual image of the forming sequences, so that the individual frames can be assigned to the corresponding clusters. Figure 1 illustrates the result of the clustering process for a forming sequence of the AA6014-S050. Overall, most of the forming sequence is attributed to the homogeneous forming phase, while also a short period is covered by the transition and necking phase. Please refer to the work in [26] for a comprehensive description of the clustering process.

### 3.5. Segmentation of the Necking Effect

While the weakly supervised approach, as described above, exclusively establishes a separation on image scale, the proposed method additionally pursues segmentation of the necking area on pixel scale. This segmentation is derived by the decoding path, which determines a pixel-wise probabilistic class affiliation by means of the softmax function that is employed on the last layer. Consequently, every pixel receives a probability of either belonging to the homogeneous or inhomogeneous forming phase, as the transition phase is not explicitly labeled. In order to realize these segmentations, binary masks that correspond to the individual images are provided during the training process. These masks are automatically generated from the extreme cases of the homogeneous and inhomogeneous phase (see Table 2) for each material with a threshold value of 99.0% of the maximum $\varepsilon_{3}$. Specifically, this value is determined such that a larger part of the image area is covered by strain localization, which ensures that the actual thinning region is included in the segmentation masks in conjunction with its neighboring pixels. As there are no localization effects in the strain distribution of the homogeneous phase, the binary masks consist exclusively of background pixels (class 0), while the structures for the individual images of the inhomogeneous phase are highlighted by foreground pixels (class 1). In order to remove single masked pixels that are not connected to the approximated necking region, a classical morphological transformation, called closing, is applied to the masks. Figure 2 illustrates three examples of the differential strain distribution together with their corresponding binary masks for three forming experiments of DP800-S050. As these examples demonstrate, the binary mask highlights a necking region including adjacent pixels, thereby forcing the network to focus on this particular region. Consequently, the network optimizes two different criteria. On the one hand, an optimal separation of the classes on image scale is pursued by employing the contrastive loss ($C_{CL}$) (cf. Equation (3)) using the features of the bottleneck layer of the encoding path, while, on the other hand, the segmentation of the localized necking region on pixel scale is derived by optimizing the binary cross entropy ($C_{BCE}$) of the decoding path:

(4) $$C_{BCE}\left(\theta_{d}\right)=-\frac{1}{N_{I}}\sum_{i=1}^{N_{I}}\left[y_{i}^{I}\log{\hat{y}}_{i}^{I}+\left(1-y_{i}^{I}\right)\log\left(1-{\hat{y}}_{i}^{I}\right)\right]$$

where $y^{I}$ and ${\hat{y}}^{I}$ denote the pixel-wise label mask and its prediction with resolution $N_{I}$. Consequently, the entire loss function is composed as

(5) $$C_{total}\left(\theta ,y,y_{1}^{I},x_{1},x_{2}\right)=\frac{1}{2}C_{CL}\left(\theta_{e},y,x_{1},x_{2}\right)+\frac{1}{2}C_{BCE}\left(\theta_{d},y_{1}^{I},x_{1}\right)$$

Within the training phase, the network learns to identify structures and critical behavior on image and pixel scale that are characteristic for the specific regions of the inhomogeneous class. Simultaneously, an optimal separation is obtained between the extreme cases of the homogeneous and inhomogeneous forming phase. Within the test phase all intermediate and unseen frames are processed by the network, so that critical regions and their development are highlighted accordingly. Therefore, the temporal as well as the spatial determination of the onset of local necking is feasible. Furthermore, the observation of the development of the necking region is provided for the entire forming process.

### 3.6. Dice Coefficient

In order to assess the quality of the segmentation method, a comparison between the estimates and the automatically generated ground truth annotations is provided by the dice coefficient [36], which enables an evaluation on pixel scale:

(6) $$\mathrm{Dice}=\frac{2\;TP}{\left(TP+FP)+(TP+FN\right)}$$

The dice coefficient determines the spatial overlap of the ground truth mask with the prediction and thus represents a metric for assessing reproducibility. The value of the dice coefficient ranges from 0 to 1, which indicates that either no or complete spatial overlap between the ground truth mask and the result of the binary segmentation is determined. The number of pixels being classified as fore- or background pixel is obtained by using a threshold value of 0.5 and are consequently being interpreted as True Positives, TP; False Positives, FP; and False Negatives, FN, per prediction.

## 4. Evaluation Experiments

The generalization and transferability of knowledge to unseen data is evaluated with a leave one material out cross-validation (LOMOCV) experiment. For this purpose, each material is held out once for the training of the network, so that this held out material can be used as a test data set for evaluation. In each case, all geometries of all included materials are used for the training of the network. Two-thirds of the sequences are used as a training set, while the remaining sequences serve as a validation set to control the training process. Consequently, for example, if training the network for DP800, the training and validation data set comprises only sequences of DX54D and AA6014. This procedure is necessary to increase the data set size and its variance, since the number of images of the necking class is very limited for all materials (cf. Table 2), and to simultaneously evaluate generalization to an unseen material or data set. The generation of the FLC proceeds analogously to the work in [26]. Consequently, the sequences of the held out material are processed by the network to generate their low-dimensional representations. The actual strain values are then determined geometry-wise by means of SMM with the described cluster procedure and three processed sequences per geometry. Subsequently, the transitions of the probability curves of the individual clusters (50% probability) are used as look-up points for the actual strain values. In addition to this evaluation on image scale, the assessment of local necking and its growth behavior on pixel scale follows the described segmentation method. In order to determine the quality of the segmentation results, the dice coefficient is employed. Consequently, a comparison of the results on image scale with the segmentation results on pixel scale is enabled, as well as a comparison with the weakly supervised FLC candidates of [26], so that the investigation weather the architectural or preprocessing changes affect the determination of the FLC is facilitated. Overall, the training process only considers the center cropped area of the strain difference distributions and a limited amount of images of the homogeneous and inhomogeneous forming phase with their respective masks (cf. Table 2). Note that # defines the number of available images per class.

Keras, a high-level API of the TensorFlow framework [37], is used for implementation of the network architecture. The experiment employs the Adam optimizer [38] for loss minimization at a learning rate of 0.00001.

## 5. Results and Discussion

**Figure 4:**
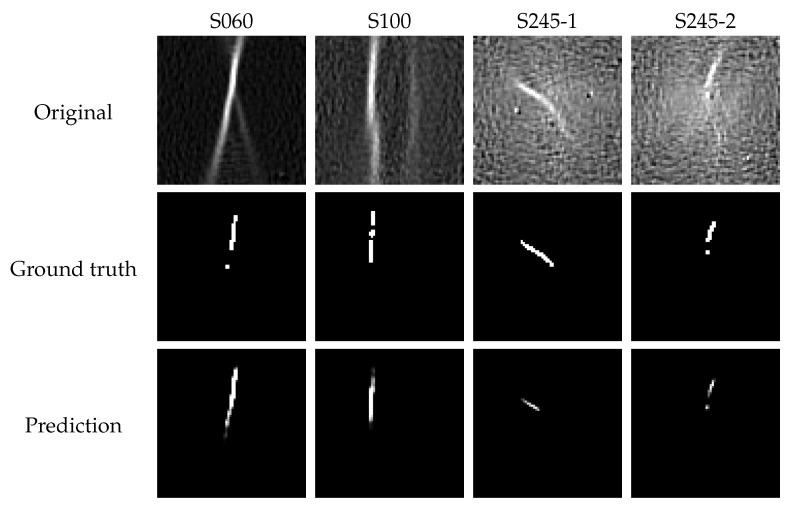
Difference strain images of AA6014 together with their ground truth annotations and predictions. Correct segmentation results are generated despite the presence of outliers in S245-1 and S245-2. In S245-2, an outlier is incorrectly included in the ground truth mask and predicted accordingly. The single pixel in the ground truth mask of S060 is a result of the mask generation process and not really an outlier, as it occurs within the necking structure. The same accounts for the discontinuity in S100.

Overall, very good dice coefficients are obtained throughout all geometries and materials, resulting in an average dice coefficient of above 0.90 among all materials in case of the foreground class, whereas a dice coefficient of 0.99 is derived for the background class. Figure 4 provides an overview of the incremental strain images together with their ground truth segmentations and predictions.

Even though only coarse segmentation masks were provided that included the localized necking plus its vicinity, the network was able to focus and emphasize the intended region. In particular, the ground truth mask of AA6014-S060 and AA6014-S100 contain discontinuities that impede the dice coefficient computation. It is difficult to argue if those discontinuities are actually correct, as comparing with the original strain distribution, the region reveals high intensities that might also be considered as foreground. Consequently, the dice coefficient only provides an indication if the network focuses on correct parts of the image, but cannot be used as a direct measure of quality, since the ground truth is only approximately correct. As there exists no explicit definition of localized necking, it is difficult to only segment the actual localized necking region automatically. However, the network learns the specific characteristics of localized necking based on the extreme cases and thus infers the necking probability to individual pixels. This is particularly well visualized for AA6014-S245-1 and S245-2, as the ground truth mask contains a larger region and thus pixels that not really exhibit necking characteristics. Consequently, the predictions only highlight few pixels with necking characteristics and thus result in lower dice coefficients.

**Figure 5:**
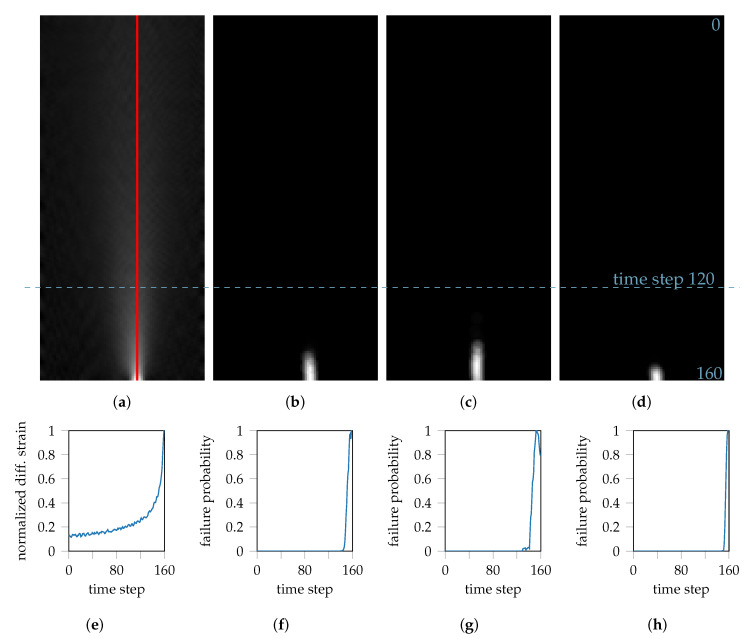
The images illustrate the temporal development of the strain distributions and their necking probabilities. The red line emphasizes the evaluation region of the line-profiles: (**a**,**e**) x-z representation of the difference images of DP800-S050-1 together with its incremental strain development as line-profile. (**b**,**f**) x-z representation of the segmented necking probability for DP800-S050-1 together with its line-profile through the maximum value. (**c**,**g**) and (**d**,**h**) x-z representation and line-profile for DP800-S050-2 and DP800-S050-3.

In order to better approximate the beginning of localized necking, the temporal development of the maximum of the difference images is considered first. This is illustrated for DP800-S050-1 in Figure 5a, together with its line profile in Figure 5e. At the beginning of the forming process, the line profile reveals a slightly increasing tendency for the gray level intensities, which increases significantly towards the end of the forming process. In the associated segmentation (cf. Figure 5b), or more precisely in the line profile through the maximum of the prediction (cf. Figure 5f), no necking probability has been predicted for the majority of the forming process. Only towards the end of the forming process, the necking probability increases fast and significantly. This deformation behavior can also be observed in the remaining repetitions for DP800-S050-2 and DP800-S050-3.

**Figure 6:**
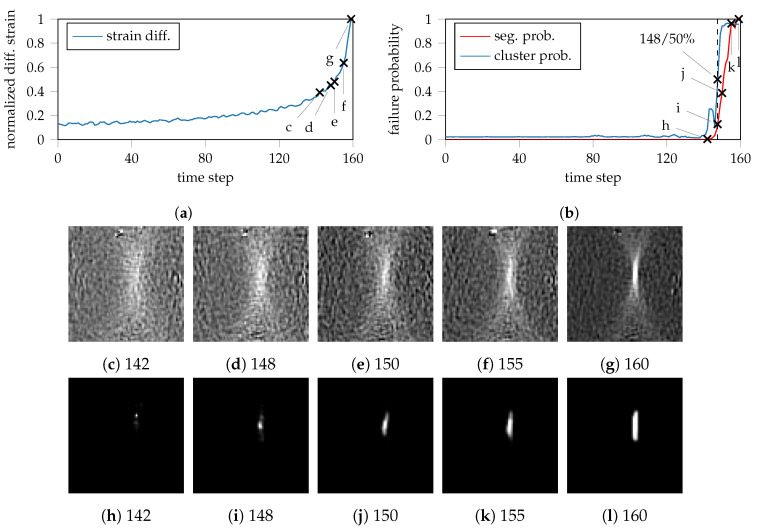
Line profiles for DP800-S050-1 during the forming process and the corresponding probability progression results for the proposed methods on image and pixel scale: (**a**) Progression of the incremental strain development through the maximum. (**b**) Corresponding probability progression through the maximum value of the prediction on pixel scale (seg. prob) together with the cluster result on image scale (cluster prob). Especially in case of the image scale approach, the effect of measurement errors or outliers is emphasized by the premature rise of the failure probability that ultimately should remain lower for a couple of more frames. (**c**–**g**) Difference strain distributions at multiple time points. (**h**–**l**) Corresponding development of the segmented area, or approximated necking region.

The in-depth investigation of DP800-S050-1 compares the critical forming period that emerges after time step 120 of the forming process (cf. Figure 5a,e), by contrasting the line profile of the difference images (cf. Figure 6a) to the corresponding line profile of the necking probability (cf. Figure 6b) to emphasize their different development. For this purpose, the difference images are compared with the predicted segmented masks at several points in time, so that the necking area and its extent are visually highlighted. The line profile of the difference images in Figure 6a reveals no significant increase up to the time point 155. The difference images at the respective points in time 142–150 (cf. Figure 6c–e) also do not exhibit a clear necking that is easy to identify. Visually one could suspect that localized necking starts at time point 150 (cf. Figure 6e), whereby this can not be recognized distinctly until time point 155 (cf. Figure 6f). At time point 160 (cf. Figure 6g), i.e., the last image of the forming process, a well-defined necking region can be identified. When investigating the corresponding predictions (cf. Figure 6b), individual pixels are already classified as belonging to the necking class at time point 142 (cf. Figure 6h), whereby the localization effect occurs at another position in the image area in comparison to the remaining predictions. Beginning with time point 148 (cf. Figure 6i), a small connected area is identified, which constantly increases until the end of the forming process at time point 160 (cf. Figure 6l). To emphasize a well-defined necking area, the predictions were spatially and temporally smoothed using a standard 3D-mean filter for image processing.

Although the necking area is clearly emphasized visually, the question arises to what extent individual pixels with low probability actually permit a reliable classification of the beginning of necking. The predicted mask at time point 148 (Figure 6i) illustrates a contiguous area classified as necking, whereby the probability of necking according to the line profile in Figure 6b reaches only 15%. Although this is considerably below the usual 50% that serves as the decision threshold in classification experiments, due to the consistency of the area and the absence of misclassified background pixels, the 15% failure probability is to be considered as a significant change, so that necking can be assumed to begin at this point in time or even slightly earlier. With these observations, generally the question arises whether the necking area should be defined on the last image of the forming sequence with respect to heuristic methods or the generation of strain paths. Although the necking area can be identified very effectively at this point, an excessive evaluation region might lead to an attenuation effect for an earlier point in time of the forming process due to the averaging of the contained strain values.

As the proposed method possesses the same capabilities as the weakly supervised approach as a result of the encoding path, it is feasible to contrast the onset of necking based on image scale with the segmentation of the necking region on pixel scale. For this purpose, the features of the bottleneck layer are again utilized, such that individual frames of the forming process are assigned to the failure classes by means of clustering using SMM. The resulting probability progression curve is additionally provided in Figure 6b and depicted by cluster probability. At time point 148, the evaluation based on the bottleneck features yields a failure probability of 50%, which corresponds to the 15% probability of the segmentation approach. The proposed methodology thus closes the gap of the weakly supervised approach, which used features of the entire image without providing an estimate of the expected necking region. With the presented method, it is thus possible to either use the whole image information, directly use the probability of the segmented area or use both evaluation options in combination. Furthermore, it can be inferred from the two curves that they begin to rise significantly at about the same time point (~145 in Figure 6b), so that an earlier or smaller quantile (<50%) should be used to define the beginning of necking in the probabilistic FLC. The difference between the two curves is particularly noticeable in the premature and temporary increase of the clustering approach. This can be attributed to the untreated outliers, which are also visible in the upper part of the difference images in Figure 6c–g.

**Figure 7:**
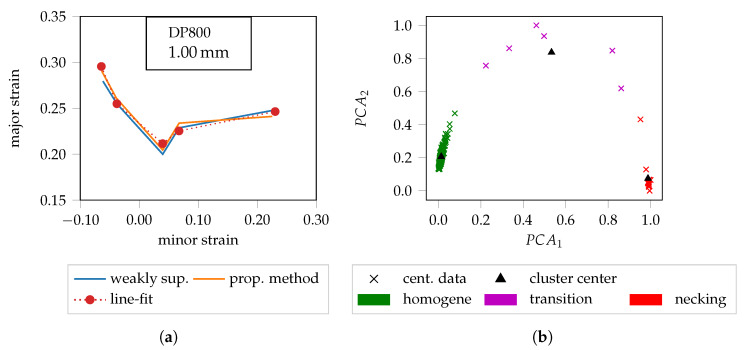
Comparison of the weakly supervised approach and the proposed method together with the impact of outliers: (**a**) DP800 FLC candidates. (**b**) Especially the transition region contains outliers that affect the cluster position.

These outliers affect the features in the bottleneck layer, so that individual samples exhibit an increased noise behavior within the clustering procedure. This is illustrated in Figure 7b and especially emphasized by the samples of the transition region. This noise behavior impairs the clustering procedure, so that the cluster centers deviate marginally and thus affect the likelihoods. A comparison of the probabilistic FLC between the weakly supervised and the cluster method based on the encoding path of the proposed method is provided in Figure 7a. Overall, for DP800, a high degree of agreement prevails, with slight deviations for the biaxial loading condition on the right sight of the curve, whereby the differences between the two FLC candidates can be attributed to the varying treatment of the outliers.

**Figure 8:**
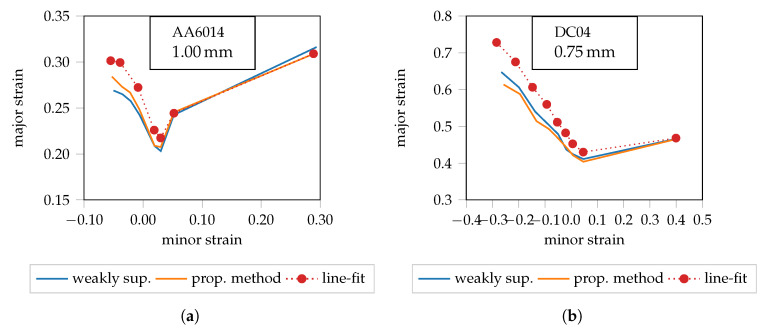
Comparison of the weakly supervised approach and the proposed method. (**a**) AA6014 FLC candidates. (**b**) DC04 FLC candidates.

In contrast to the weakly supervised method, the data is now preprocessed using robust scaling. As a result, there is no saturation of the extreme values, so that the necking character is maintained both in the presence and absence of outliers. Consequently, the FLCs of the proposed method deviate marginally from the weakly supervised results (cf. Figure 8a,b). In order to further improve the interpretation of the results, the FLCs determined by the “line-fit” method [6] are also provided in the diagrams. Note that the intersection between the transitions and the necking phase (cf. Figure 1a) is used to generate the FLCs of the proposed method. This corresponds to the 50% quantile of the local necking phase (cf. Figure 6b). To improve visibility, additional quantiles (cf. [17]) are not presented. The 50% quantiles of both machine learning approaches lie below the FLCs of the “line-fit” method for AA6014 and DC04. Conversely, the FLCs for DP800 coincide, so that in this case it can be inferred that the “line-fit” method tends to overestimate the forming capacity as highlighted in Figure 6.

## 6. Summary

The proposed method incorporates all the advantages of the weakly supervised approach of Jaremenko et al. [26], which comprises the data-driven learning of appropriate features and the time- and location-independent determination of the onset of localized necking. Consequently, using the features of the bottleneck layer provides the possibility to determine the temporal onset of localized necking based on image scale features. Additionally, the spatial determination of localized necking is realized by the decoding path of the network, such that a probabilistic assessment of the necking region and its growth behavior on pixel scale is feasible. As a consequence, it is possible to generate strain paths by only evaluating the specific area that is actually involved in the necking process rather than heuristically specifying the necking area on the last image of the forming process. Furthermore, it was determined that it would be reasonable to consider a lower failure quantile of the probabilistic FLC (<50%) in process design that defines the onset of localized necking. Future work should incorporate temporal information to facilitate a smooth determination of the necking region without the necessity of mean filtering. However, in order to provide a final evaluation of the presented methodology, it is essential to produce real components and to examine their quality by means of metallographic investigations.

## Figures and Tables

**Table 1:** Properties of the investigated materials.

Material	t_0_ [mm]	n	YS [MPa]	TS [MPa]	UE [%]	r_0_	r_90_
DX54D	0.75, 2.00	0.23	164–170	297–322	22-23	1.80	2.22
DP800	1.00	0.16	465	775–797	14–16	0.76	0.90
AA6014	1.00	0.24	140–143	239–244	21–23	0.69	0.67

**Table 2:** Database per material with process parameters.

Material (Thickness mm)	# Images/ # homog./# neck.	Frequency [Hz]	Punch Velocity [mm/s]	Available Geometries
DX54D (2.00)	80/20/5	20	1.5	S030, S060, S080, S100, S125, S245
AA6014 (1.00)	55/15/3	15	1.0	2S050, S060, S080, S100, S110, S125, S245
DP800 (1.00)	160/20/3	40	1.0	S050, S060, S110, S125, S245
DX54D (0.75)	160/20/5	40	1.0	2S050, S060, S070, S080, S090, S100, S110, S125, S125, S245
